# Role of bulk nanobubbles in removing organic pollutants in wastewater treatment

**DOI:** 10.1186/s13568-021-01254-0

**Published:** 2021-06-28

**Authors:** Jiajia Wu, Kejia Zhang, Cheng Cen, Xiaogang Wu, Ruyin Mao, Yingying Zheng

**Affiliations:** College of Civil Engineering and Architecture, Zhejiang University, Hangzhou, 310058 China

**Keywords:** Bulk nanobubbles, Flotation, Aerobic biological treatment, Free radicals, Organic pollutants

## Abstract

The occurrence of a variety of organic pollutants has complicated wastewater treatment; thus, the search for sustainable and effective treatment technology has drawn significant attention. In recent years, bulk nanobubbles, which have extraordinary properties differing from those of microbubbles, including high stability and long residence times in water, large specific surface areas, high gas transfer efficiency and interface potential, and the capability to generate free radicals, have shown attractive technological advantages and promising application prospects for wastewater treatment. In this review, the basic characteristics of bulk nanobubbles are summarized in detail, and recent findings related to their implementation pathways and mechanisms in organic wastewater treatment are systematically discussed, which includes improving the air flotation process, increasing water aeration to promote aerobic biological technologies including biological activated carbon, activated sludge, and membrane bioreactors, and generating active free radicals that oxidise organic compounds. Finally, the current technological difficulties of bulk nanobubbles are analysed, and future focus areas for research on bulk nanobubble technology are also proposed.

## Key points

- Bulk nanobubbles can enhance the flotation efficiency to remove organics like grease.
- Oxygen mass transfer rate is accelerated for microbials biodegradation processes.
- The collapse of bulk nanobubbles forms free radicals which promote oxidation process.

## Introduction

Nanobubbles are recognised as spherical packages with diameters of less than 1 μm that exist at the solid–liquid interface or dispersed in the liquid medium (Alheshibri et al. 2016). They can be classified into solid–liquid interface nanobubbles (surface nanobubbles) and bulk nanobubbles according to their morphologies and locations. Surface nanobubbles are pancake-like bubbles adsorbed at the solid–liquid interface with a height above the substrate of 20–30 nm and a radius of curvature of the order of 100 nm (Tyrrell and Attard 2001), while the morphology of the bulk nanobubbles appears as a complete sphere with nanometre-sized dimensions less than 1 μm. Both the research history and methods used to study the two types of nanobubbles are relatively independent systems. The discrepancy in their morphology also resulted in great differences in the production methods and applications of the two types of nanobubbles. At present, with the advancement of research, several methods, including direct immersion, temperature changes, electrochemical reactions, and ethanol–water exchange, have been applied to generate surface nanobubbles (Qiu et al. 2017), which are used for surface cleaning, and micro-scale pipeline design; however these technologies are not yet well developed. Bulk nanobubbles produced by the electrolytic method (Hao et al. 2020), the porous membrane method (Ulatowski et al. 2019), sonication cavitation (Ulatowski et al. 2019), and hydrodynamic cavitation (Oliveira et al. 2018), on the other hand, play a predominant role in water treatment.

Unlike ordinary microbubbles (diameter less than 50 μm) or conventional large bubbles (diameter larger than 1 mm), bulk nanobubbles exhibit peculiar physical properties which have drawn extensive attention for applications in bioremediation, agriculture, and biomedicine, and particularly show great technological advantages and prospects in the field of wastewater treatment. Due to the shortage of water resources and serious water pollution, wastewater treatment has become a focus of social development. Recently, bulk nanobubbles have been used extensively in the treatment of oily wastewater, coke wastewater, printing and dyeing wastewater (Bui and Han 2020), acrylic fibre wastewater (Zheng et al. 2015), and heavy metal ion wastewater (Kyzas et al. 2019), etc. With the huge aforementioned applications of bulk nanobubbles, it is anticipated that bulk nanobubbles can be a potential driver for strengthening technologies in water treatment and thus play a major role in industrial applications relative to surface nanobubbles. Organics, such as taste and odor compounds, which are extremely complex and considered earthy-musty (Zhou et al. 2021), influence people’s life to a great extent (Cen et al. 2020) and increase the difficulty and cost in treatment processes. Exponential researches have been carried out to explore the development of bulk nanobubble technology to deal with organic pollutants in wastewater since it’s environment-friendly and has no secondary pollution. However, it can be found that large amounts of previous studies about bulk nanobubble-based flotation method focus on the removal of metal ions, little researches pay special attention to the organic matter, although the flotation process is the common step of water treatment in plants, which deserves the concentration in-depth. On the other hand, the aerobic biological methods are extensively used for water treatment, but there is rare review particularly highlighting the improvement of organics biodegradation efficacy via combination of bulk nanobubbles and aerobic biological technologies, meanwhile, clarifying the mechanism to promote more profound study and application.

The major objective of this study is to comprehensively characterise the fundamental properties of bulk nanobubbles and provide insights on the current application status and mechanisms of this technology for treating wastewater containing organic pollutants, aerobic biological methods integrated with bulk nanobubbles are emphasised. In the end, the problems including what is unknown about organic pollutants in wastewater treatment which demand prompt solution and future perspectives in this field are addressed simultaneously.

## Fundamental properties

### High stability

The earliest investigation of bulk nanobubbles can be found in an article published by Johnson and Cooke (1981). They reported that shear in seawater could generate gas-filled nanometre-sized bubbles which were stable for up to 24 h as a result of the existence of surface films formed from naturally present surfactants. However, according to classical thermodynamics, the additional pressure that exists in a spherical bubble in a liquid can be characterised by the Young–Laplace formula equation [Eq. (1); Liu and Cao 2016]:

1 $$\Delta P = \frac{{2\sigma }}{r}$$

where ΔP is the pressure difference between the inside and outside of the bubble, also called Laplace pressure; σ is the surface tension of the bubble/liquid interface; and r is the bubble radius. As bubbles shrink, the pressure inside will correspondingly increase, and as gas tends to dissolve into the liquid, this will cause the bubble collapse. For instance, the internal pressure of a bubble with a radius of 100 nm is as high as 1.5  ×  10^6^ Pa; when the surface tension is 72 m N/m and the liquid pressure is 10^5^ Pa (Gurung et al. 2016), calculation results suggest that nanobubbles with a diameter of 100 nm can only exist in water for 10 μs (Ljunggren and Eriksson 1997; Nirmalkar et al. 2018). Nevertheless, in the last ten years, during which bulk nanobubbles have been studied extensively (Alheshibri et al. 2016), with the advancement of nanobubble tracking technology such as laser diffraction techniques and other detection methods, additional evidence has verified that nanobubbles can stably exist in liquid media for a long time. This can be attributed to their small size and high interface potential, as described below.

After ordinary large bubbles are generated, they will rapidly move upward by buoyancy and disappear on the water surface (Demangeat 2015), while bulk nanobubbles can stay in water for days or even months (Azevedo et al. 2016; Liu et al. 2013). The rising velocity of a bubble is highly associated with its size, the rising speed and residence time can be slower and longer respectively with the bubble radius decreases. One calculation demonstrates that the rising velocity of nanobubbles with a radius of 50 nm can be as low as 2.7 nm/s (Alheshibri et al. 2016). The effect of buoyancy on nanobubbles is negligible; thus, bulk nanobubbles have a longer lifespan in water than large bubbles. Nirmalkar et al. (2018) reported that bulk nanobubble suspensions were stable over periods of several months, during which the mean diameter remained constant, suggesting the absence of significant bubble coalescence, bubble breakage, or Ostwald ripening effects (Nirmalkar et al. 2018). Incidentally, Weijs et al. (2012) provided insight into the reason for the stability using molecular dynamics, showing that nanobubbles in a cluster of bulk nanobubbles protect each other from diffusion by a shielding effect (Weijs et al. 2012).

In addition, bulk nanobubbles have a high interface potential. The potential difference caused by the surface charge of a bubble is often characterised by the zeta potential, which is a physical property exhibited by particles in suspension, that measures the electrostatic repulsion or attraction between particles and bubbles (Parmar and Majumder 2013). The zeta potential effectively predicts long-term stability in a colloidal system. If all the particles in suspension show a high zeta potential (negative or positive), they tend to be stable for a long time; otherwise, they are apt to aggregate and coalesce (Gurung et al. 2016). The surface of nanobubbles adsorbed with negatively charged OH^−^ ions is called the surface-charged ionic layer which electrically attracts positively charged H_3_O^+^ ions and consequently forms an electric double layer (Boshenyatov et al. 2019). The electrostatic repulsion forces caused by the overlap of electric double layers among neighbouring bubbles provide resistance against bubble coalescence; hence, the nanobubbles show unexpected stability and durability (Calgaroto et al. 2014). According to a theoretical model where conventional Young–Laplace equation was modified shown as equation [Eq. (2)–Eq. (5); Satpute and Earthman 2021]:

2 $$p_{{int}} + p_{{rep}} = p_{{ext}} + p_{{st}}$$

3 $$p_{{rep}} = \frac{{\sqrt 3 k_{e} \sigma ^{2} }}{2}$$

4 $$\sigma = \frac{{\varepsilon _{0} \varepsilon \xi }}{\lambda }$$

5 $$\lambda = \sqrt {\frac{{\varepsilon _{0} \varepsilon k_{B} T}}{{{\text{2}}N_{A} Iq^{{\text{2}}} }}}$$

where p_int_ is the internal pressure, p_rep_ is the Maxwell pressure due to the repulsion between the negative ions adsorbed onto the bubble surface, p_ext_ is the external pressure, p_st_ is the pressure due to surface tension, k_e_ is the Coulombic constant for the fluid (1.1 × 10^8^ N m^2^ C^−2^), σ is the surface charge density of the bubbles calculated from the zeta potential using an equation from the Debye-Hückel theory of electrical double layers, ε_0_ is the permittivity of free space, ε is the dielectric constant of the fluid, λ is the Debye length, ξ is the zeta potential, I is the bulk concentration of the stabilizing ions, i.e. the OH^−^ which is 10^−4^ mol m^−3^ at pH 7, T is temperature, k_B_ is Boltzmann’s constant, and N_A_ is Avogadro’s number.

The model successfully elucidates repulsive forces between OH^−^ on the surface are sufficient to ultimately balance the surface tension forces at nanobubble sizes, thus, making nanobubbles exhibit great stability. As shown in previous literatures, zeta potential values change with the pH of the medium vary, exhibiting a tendency that the negativity of zeta potential increases with solution pH increase which results in greater bubble surface charge and make bubbles more stable due to electrostatic repulsion (Meegoda et al. 2018). Nanobubbles generally show high negative zeta potential owing to the excess of hydroxide ions (OH^−^) relative to hydrogen ions (H^+^) at the gas–water interface of bubbles under wide range of solution pH, but nanobubbles formed in acidic environments always show lower stability compared with in neutral or alkaline conditions because a lack of OH^−^ are required to stabilize the electric double layer (Nirmalkar et al. 2018), the zeta potential of nanobubbles generated in strong acidic medium below pH 3 tends to be positive and low which causes higher possibility for bubble coalescence (Zhang et al. 2020). A magnitude of the zeta potential of 30 mV is regarded as the critical value that can produce a great repulsion force and contribute to the persistence of nanobubbles (Ushikubo et al. 2010).

As of now, various theoretical models have been proposed to interpret the stabilization mechanism of bulk nanobubbles including electrostatic repulsion model (Yasui et al. 2018), shielding effect theory (Weijs et al. 2012), skin model (Yount 1979), particle crevice model (Atchley and Prosperetti 1989), dynamic equilibrium model (Yasui et al. 2016). Nevertheless, the unexpected stability of bulk nanobubbles needs to be further studied to obtain a unified conclusion in the future.

### High gas–liquid mass transfer efficiency

There is a gas–liquid interface around bulk nanobubbles in water which leads to compression of the bubbles and thus continuous shrinking of the bubbles during their rise to the water surface and exhibition of a self-pressurisation effect. The rate of gas diffusion from the high-pressure region to the low-pressure region is proportional to the pressure gradient (Li et al. 2014), and the mass transfer rate from the inside of the bubble to the surrounding liquid is accelerated as the bubble shrinks. Likewise, bulk nanobubbles show a large specific surface area because the surface area of the bubble is inversely proportional to the radius of the bubble (Kim et al. 2018). For example, the total surface area for a volume of 1000 bubbles with a diameter of 5 mm diameter is 0.0785 m^2^ (total volume  =  6.54  ×  10^−5^ m^3^), while for the same volume of bubbles with a diameter of 100 nm (volume/bubble = 5.24 × 10^−22^ m^3^), the total surface area is much higher, reaching 3925 m^3^, when the number of bubbles is equal to 1.25 × 10^17^ (Batagoda et al. 2019). As described previously, more gas can dissolve into water through the bubble interface, and the efficiency of the gas transfer to the liquid phase is improved effectively owing to the self-pressurisation effect of the bulk nanobubbles, long residence times and large specific surface areas. Until the internal pressure of bubbles reaches a certain limit value they will collapse and disappear at last.

### Ability to generate free radicals

Several studies have shown that strong ultrasound and hydrodynamic cavitation can cause the collapse of nanobubbles and generate free radicals (Masuda et al. 2015). During the shrinkage of nanobubbles, the electric charge density accumulated in the electric double layer increases rapidly. Because of the drastic change in the gas–liquid interface at the moment of bubble collapse, the high concentration of ions aggregated on the interface instantly releases the accumulated chemical energy. Meanwhile the entrapped water molecules are subjected to extremely high temperatures and pressures when they are forcefully compressed by dynamic stimuli (Krishnan et al. 2006), which results in cleavage that produces various reactive oxygen species (ROSs), including hydroxyl radicals (·OH), superoxide anion radicals (O_2_·^−^), and singlet oxygen (^1^O_2_) (Atkinson et al. 2019; Liu et al. 2015; Lyu et al. 2019). Several studies have applied electron spin resonance or the action of free radical inhibitors to verify their existence. Michailidi et al. (2020) used electron paramagnetic resonance based on 5,5-dimethyl-1-pyrroline N-oxide (DMPO) spin trapping to quantitatively analyse free radicals in air-nanobubble and oxygen-nanobubble systems. At a concentration of 100 mM of DMPO and on the basis of a calibration curve, a total spin concentration of about 1.5 μM in the air-nanobubble system was inferred (Michailidi et al. 2020). Wang et al. (2020) used 2-propanol, benzoquinone (BQ), and NaN_3_ as scavengers to trap ·OH, O_2_·^−^, and ^1^O_2_, respectively, to identify the possible reactive species that induced the photodegradation of oxytetracycline and concluded that ·OH, O_2_·^−^, and ^1^O_2_ could coexist in nanobubble solutions, while ·OH played a predominant role in the oxidation reaction (Wang et al. 2020). Liu et al. (2016) confirmed the existence of ·OH using a sensitive fluorescent probe, APF, in oxygen-nanobubble water and quantified the amount in submicromolar order of magnitude (Liu et al. 2016). The powerful oxidation function of these reactive oxygen species is gradually being considered for application in advanced oxidation processes to treat persistent organic pollutants in wastewater treatment (such as polychlorinated biphenyls and phenolic halogenated compounds) and also be used for disinfection of pathogens.

It is precisely due to the peculiar advantages mentioned above, the bulk nanobubbles are drawing more attention in wastewater treatment field, and show promising potential in improving common processes to remove organic matter. Based on previous researches, it can be concluded that bulk nanobubbles can remove organic pollutants in water through various pathways, approximately including: (1) improvement of the air flotation process to remove fats, oils, low-density organic suspended solids, and colloids; (2) promotion of aeration in water to strengthen the conventional biodegradation processes consisting of biological activated carbon filters, activated sludge and membrane bioreactors; (3) generation of free radicals to oxidise and degrade organic compounds that are difficult to biodegrade, which will be elucidated deeply in upcoming sections.

## Mechanism and improvement of flotation

Flotation is regarded as the most reliable and practical method for removing suspensions containing fats, oils, low-density organic suspended solids, and colloids (Colic et al. 2007). It’s extensively employed in removing various organic matters in the pre-treatment unit of wastewater treatment system to decrease the load of the subsequent treatment processes. The separation mechanism is attributed to the adsorption of gas bubbles on the surface of the fine suspended particles, thereby forming bubble-particle aggregates, which significantly decreases the gravity of the contaminants. The particles thus rise to the water surface more easily where they can be scraped off to accomplish solid–liquid or liquid–liquid separation, achieving removal objectives (Zimmerman et al. 2011). The formation of bubble-particle aggregates depends on lots of factors, including the ratio of bubble/particle size, concentration, etc., among which gas dispersion parameters are regarded as the most important in the separation. Conventional air flotation methods are effective for particles with a narrow size range, with the flotation efficiency significantly reduced beyond the optimal size range (Fan et al. 2010). Low flotation rates are ascribed to the possibility of bubble-particle collisions, and the detachment of bubble-particle is lower and higher, respectively, when the particle size is larger or smaller (Tao 2005). Experimental evidence suggests that the presence of nanobubbles broadens the range in the flotation particle size, increases the surface hydrophobicity of particles, and improves froth flotation efficiency (Fan et al. 2010; Sobhy and Tao 2018; Tao and Sobhy 2019). The overall efficiency of flotation is determined by three consecutive steps: bubble-particle collision, attachment, and detachment. Various models and studies have shown that the need to reduce the bubble size is highly related to an increase in the possibility of collision between bubbles and particles, and the efficacy of flotation in the separation process of pollutants can be substantially improved owing to the small size of nanobubbles and the presence of opposite surface charges, which increase the probability of collision and attachment between particles and bubbles (Luttrell and Yoon 1992; Xiao et al. 2018). Mishchuk (2005) carried out a theoretical analysis of interparticle interaction in a nanobubble-containing system and reported that the appearance and disappearance of nanobubbles affected both mechanical and other characteristics of the liquid layer between macrobodies, including density, fluidity of liquid, and conditions of rupture of thin non-homogeneous film. Their results further showed that nanobubbles in the gap between two similar particles increased their attraction, resulting in an improvement of the aggregation formation (Mishchuk 2005). Nazari et al. (2019) investigated the influence of the bubble size distribution on the flotation behaviour of coarse quartz particles and found that the amount of adsorbed dodecyl amine on the surface of the quartz particles in the presence of nanobubbles with a diameter of 171 nm was greater than that in the presence of nanobubbles with sizes of 110 and 293 nm and in common air bubbles (Nazari et al. 2019). Tsai et al. (2007) studied nanobubble flotation technology (NBFT) with a coagulation process for treating laboratory-scale and pilot-scale chemical mechanical polishing (CMP) wastewater, and their experimental results showed that compared with the traditional coagulation/flocculation process, the combination of NBFT and coagulation technology elevated the turbidity removal efficiency of wastewater by 40% (Tsai et al. 2007). Furthermore, Calgaroto et al. (2016) removed decyl trimethyl ether amine by nanobubble flotation. Nanobubbles adhered to the amine precipitation formed by amine at pH 10.8, entrained inside the floc through flotation separation, and the removal rate of amine reached 80%, indicating that the application of nanobubbles in flotation technology has the potential to treat residual amine-containing wastewater (Calgaroto et al. 2016). Additionally, Zhou et al. (2019) investigated the adsorption behaviour of bulk nanobubbles produced in the hydrodynamic cavitation on muscovite surfaces in the presence of dodecylamine (DDA) (Zhou et al. 2019). The results demonstrated that nanobubbles were largely coated with DDA and indeed absorbed on the muscovite surfaces, stabilised probably by three-phase line pinning, leading to extensive enhancement of muscovite flotation performance. Etchepare et al. (2017) investigated the separation of emulsified crude oil in saline water with microbubbles (30–40 mm) and nanobubbles (150–350 nm); the input of isolated nanobubbles (3 × 10^8^ nanobubbles/mL) after flocculation with 1 and 3 mg/L Dismulgan increased the hydrophobicity of the aggregates, promoted the adhesion between bubbles and oily flocs, and the removal efficiency of the flotation was enhanced from 73 to 84% and from 92 to 95%, respectively (Etchepare et al. 2017). Based on the above research the application of bulk nanobubbles improves the flotation efficiency, leading to better removal of substances such as grease.

## Aerobic biological treatment

For organic wastewater with good biodegradability, biological treatment methods, including biological activated carbon filtration, biological activated sludge, and membrane bioreactors (MBRs) which consume less energy and are environmentally friendly, compared with other physical and chemical technologies like electrochemical methods or which need extra addition of chemicals, have become a preferential treatment technology. Biological methods decompose toxic and harmful chemicals and certain components that exceed the standard in water using the metabolism of microorganisms. The main limitation and costs of aerobic biological processes emerge from aeration, sludge treatment, and membrane fouling.

Oxygen plays a critical role in the metabolism of aerobic organisms and the biochemical reaction substrate for oxidative degradation of pollutants, which is directly related to the removal effect of biodegradable organic matter during wastewater treatment processes, not only in biological activated carbon filtration but also in activated sludge and membrane bioreactors. Therefore, the mass transfer rate of oxygen needs to be accelerated in order for the dissolved oxygen concentration in water to be increased to promote the degradation of organics. The vital role of aeration in oxygen delivery has been widely acknowledged. Traditional mechanical aerators or diffusers consume large amounts of electrical energy, and the mechanical maintenance costs are high (El-Zahaby and El-Gendy 2016), while the oxygen transmission efficiency is extremely unsatisfactory, which is not conducive to energy conservation and consumption reduction. Nanobubbles showing negligible buoyancy and long residence times in water makes it possible to diffuse oxygen more effective. Zhang et al. (2018) studied the restoration of lake sediments, and their results indicated that the supply of oxygenated nanobubble-modified zeolite/soils, which was achieved via exposing the materials to oxygen supersaturating ambient conditions and then reloading O_2_ in the particle micropores, to the anoxic lake sediments could sustain dissolved oxygen at concentrations greater than 6 mg/L for more than 6 months (Zhang et al. 2018). Li et al. (2013) compared the influence of micro-nano bubbles in groundwater bioremediation and found that the mass transfer rate of oxygen in micro-nano bubbles was 125 times faster than that of large bubbles, and the dissolved oxygen was 16 times more durable than the latter. Additionally, the study explored the effect of nanobubbles on aerobic organism biodegradation of pollutants in water, and the results showed that the oxygen utilisation rate and volumetric mass transfer coefficient of the nanobubble-aerated synthetic wastewater treatment device were almost twice those of conventional bubble aeration devices, and the retention time for the degradation of organics in the nanobubble aeration unit was less than half of that used in traditional systems. Based on these results, it was suggested that nanobubbles could promote aerobic biodegradation process to clean up the contaminated groundwater (Li et al. 2013). Recently, Xiao and Xu (2020) attempted to provide more oxygen in aerobic biofilm systems to improve their performance and reduce the aeration costs for wastewater treatment (Xiao and Xu 2020). The experiments indicated that nanobubbles offer a superior oxygen supply capacity and 1.5 times higher oxygen transfer efficiency compared with large bubbles, which promoted the growth of the biofilm and achieved better removal efficiencies of chemical oxygen demand and ammonia. Furthermore, nanobubble aeration resulted in energy saving of approximately 80%. Rameshkumar et al. (2019) generated nanobubbles in four systems, including tap water, pond water, domestic wastewater, and industrial wastewater, to explore the influence of nanobubbles on water treatment efficiency. Surprisingly, there was a dramatic increase in the dissolved oxygen concentration after water treatment in all four systems, where the dissolved oxygen increased to 2 times and 1.5 times that of tap water and pond water respectively. Meanwhile, it was noticed that the chemical oxygen demand decreased from 399 mg/L before treatment to 4 mg/L below the detection limit in domestic wastewater, and from 4520 mg/L to 124 mg/L in industrial wastewater, proving the distinct effectiveness of nanobubbles in water treatment (Rameshkumar et al. 2019). As mentioned above, there is no doubt that nanobubbles have promising application prospects in the enhancement of aeration and microbial growth for biodegradation.

### Biological activated carbon

Adsorption supplemented with biodegradation is recognised as the predominant mechanism contributing to the removal of dissolved organic chemicals (DOC) in the biological activated carbon (BAC) filter process. Activated carbon is widely applied to remove or control unpleasant taste and odor issues and organic contaminants such as chloroform and trihalomethanes in water, as well as various kinds of pollutants, including aromatic compounds, hydrocarbons, detergents, soluble dyes, and phenols, owing to its high porosity and extensive internal surface area (Rattier et al. 2012). Over time, the adsorption sites of activated carbon are saturated with organics, leading to the loss of the initial effectiveness, accompanied by the development of a biologically active biofilm due to bacteria inhabiting the pores of the carbon where they form a microbial community. This ensures that the carbon keeps removing DOC through metabolic activity and the biological oxidation responses to the removal action in this stage. Based on this, biodegradation in BAC filters should be emphasised. As stated previously, nanobubbles significantly improve the aeration and oxygen mass transfer efficiency in aerobic biofilm systems for microbial biodegradation. Noteworthy is also that bulk nanobubbles act not only on biodegradation, but also on adsorption. Kyzas et al. (2019) investigated the effect of nanobubbles on lead ion adsorption by activated carbon in water and verified that the adsorption capacity was approximately similar in the presence or absence of nanobubbles; however, the adsorption process was impressively accelerated by 366%, which is expected to reduce the adsorption equilibrium time and subsequently increase adsorption–desorption recycling; the mechanism derived from these results is that nanobubbles attract charged particles onto their interface that assist the diffusion and penetration of lead ions into the activated carbon pores (Kyzas et al. 2019). However, the influence of bulk nanobubbles on the adsorption of organic compounds on activated carbon and the underlying mechanism still need to be explored.

### Activated sludge

The activated sludge system consists of biodegradation and sedimentation processes that are carried out in aeration and sedimentation tanks, respectively. The aeration tank is filled with well-mixed aerated wastewater for long time periods to achieve biological conversion of dissolved and colloidal substrates into stabilised and new biomass cells. This process is performed by a great diversity of microbes in the presence of oxygen. The effect of nanobubble aeration on the two most common microbial aggregates, activated sludge and biofilms, was evaluated by Xiao et al. (2021), and their results indicated that the utilisation of nanobubbles successfully supplied extra oxygen for microbial aggregates and achieved a 10.58% increment in total nitrogen removal. Furthermore, the structure of the microbial aggregates was improved, showing that extracellular protein and polysaccharides, increased to the highest levels of 3.40 times and 1.70 times in the biofilm and activated sludge, respectively, with the thickness of the biofilm and activated sludge floc size increasing. This results suggested that the nanobubbles optimised the distribution of functional microbes and their metabolic pathways by speeding up the structural development of the microbial aggregates, which simultaneously resulted in an adaptive process in the microbial aggregates, especially for activated sludge exhibiting a negative impact on settleability when under a low addition ratio of nanobubbles, while it became positive if the ratio exceeded 50% (Xiao et al. 2021). Sludge disintegration and solubilisations are tricky problems in the long-term performance of activated sludge processes. Sludge ozonation was shown to have a promising effect on reducing waste sludge volume. A sludge floc is composed of a complicated microbial community that includes bacteria, protozoa, and metazoans, and forms microbial aggregates called flocs bound in company with extracellular polymeric substances, such as proteins, carbohydrates, humic acids, and other organic molecules (Semblante et al. 2017). Before ozone reacts with bacterial cells, it is first consumed by dissolved organic matter and extracellular polymeric substances. The size of sludge flocs varies from tens of microns to several hundred microns, while the gaps are approximately 20 μm. As ozone carriers, conventional microbubbles are too large to penetrate the gaps of sludge flocs, limiting the ability of ozone to reduce the sludge volume, whereas the diameter of the nanobubbles is two orders of magnitude smaller than the gaps. Thus, ozone consumption outside the flocs is avoided, while ozone plays key role in killing bacteria. Hashimoto et al. (2021) employed the death ratio of bacteria in sludge as an indicator of the effectiveness of sludge reduction and compared the ability to supply ozone for sludge reduction of nanobubbles and microbubbles; the results demonstrated that the ozone dose required to reach a death ratio of 80% was 15 mg-O_3_/g-MLSS in a system where ozone was carried by nanobubbles versus 25 mg-O_3_/g-MLSS when ozone was supplied by spiral, liquid-type microbubbles. In addition, the depth of the dead cell layer from the surface to the interior of the sludge floc was larger in a system with nanobubbles than in one lacking nanobubbles at the same rate of ozone consumption, indicating that nanobubble ozone carriers improved the efficiency of sludge reduction (Hashimoto et al. 2021).

### Membrane bioreactor

Aerobic MBRs, which utilise aerobic digestion in combination with membrane filtration, are known as a substitute for conventional activated sludge treatment, where the secondary clarifier is replaced by a membrane in order to alleviate the settleability problem of undesired biomass formation. Its advantages are good effluent quality, a small footprint to reduce the total land usage, higher volumetric loading rates, less sludge production, etc., Terfasa (2017) calculated the value of the decay rate of the biomass in a nanobubble system and found that it was almost three times faster than that of traditional bubble supported systems, indicating that nanobubbles decrease excess sludge generation in aerobic treatments (Terfasa 2017). However, apart from the need to improve aeration to increase digestion efficacy, MBRs also suffer from heavy membrane biofouling issues in organic wastewater treatment that impedes operational flexibility and causes a significant increase in maintenance costs (Iorhemen et al. 2016). Sustainable membrane fouling mitigation strategies in MBRs must thus be considered. Membrane filtration was tested in both a conventional and a nanobubble-supported system in Terfasa (2017), showing that the membrane filtration flow rate decreased with time for both systems, but dropped more quickly for the conventional system. Moreover, the cumulative filtered volume of the nanobubble-supported system was enhanced faster than that of the conventional system, which can be attributed to the reduction of sludge, which blocks the membrane pores, due to the presence of nanobubbles (Terfasa 2017). Ghadimkhani et al. (2016) attempted to clean the fouled membrane surface by feeding air nanobubbles into the membrane cells using humic acid as a simulated organic foulant; the results showed that the permeate flux recovered to its initial level, indicating that the nanobubbles dramatically unclogged the pores of the membrane, which was ascribed to the fact that ·OH bonds with the organic foulants and decomposes organic matter (Ghadimkhani et al. 2016).

The application of bulk nanobubble-integrated aerobic biological technologies is capable of facilitating the biodegradation of organics, but it has not yet achieved industrial-scale application, which is an important topic for future research. Furthermore, it should also be considered whether the Brownian motion of nanobubbles and high levels of heat and shock waves generated from nanobubble bursts cause damage to the cells of the microorganisms.

## Advanced oxidation

### Bulk nanobubble-based advanced oxidation

As mentioned previously, several studies have recognised that bulk nanobubbles are capable of producing ·OH, which has a strong oxidation ability and is nonselective to almost all kinds of organic contaminants when bubbles burst, and can thus play an important role in treating wastewater-containing organic compounds that are resistant to conventional biological treatment through hydrogen abstraction, radical–radical reactions, electrophilic addition and electron transfer. Traditionally, ·OH is generated by large amounts of energy and chemically intensive processes, for instance, UV irradiation, hydrogen peroxide oxidation and Fenton oxidation; thus, nanobubbles can be an encouraging innovative alternative. Bui and Han (2020) utilised three nanobubble systems (nanobubbles, ultrasonic nanobubbles, and nanobubbles/H_2_O_2_) to degrade dark green Rit dye, and verified the production of ·OH in the system by detecting the methane sulfonic acid spectrum. The study found that ·OH generated in the system with coexisting nanobubbles and H_2_O_2_ would further react with H_2_O_2_ to form HO_2_· and O_2_·, which destroy the dye molecules and obtain a good decolourisation rate; in this system, the decolourisation rate of Rit dyes reached more than 90% after 60 min (Bui and Han 2020). Besides, electrostatic attraction was proposed to drive the decolourisation of Rit dye in the nanobubble systems, except for the function of reactive species. The results confirmed that colour removal efficiency could be improved under two conditions when the charges of the nanobubbles and dye solution were opposite, and the magnitude of the surface charge of the nanobubbles was almost similar to that of the dye in the solution. Tasaki et al. (2009) investigated the effect of an 8 W low-pressure mercury lamp on the decomposition of sodium dodecyl benzene sulfonate (SDBS) in the presence of nanobubbles (720 nm diameter). Degradation experiments were performed using an ozone lamp (185–254 nm) with and without nanobubbles. The experimental results showed that under 185–254 nm irradiation, the oxidation and mineralisation rates of SDBS were significantly improved when combined with oxygen nanobubbles, and SDBS was effectively removed in an integrated nanobubbles/vacuum ultraviolet (VUV) system. The post-SDBS oxidation rate was 99.8%, and the total organic compound (TOC) removal rate reached 76.8% after 24 h (Tasaki et al. 2009). Wang et al. (2020) added oxygen nanobubbles to photo-reaction system to improve the photodegradation efficiency of oxytetracycline under visible light irradiation. The results presented that the photodegradation efficiency increased to 60% in the oxygen nanobubbles water after 4 h reaction and even reached to 98% when adjust the solution pH to 11.0, while that of ordinary aeration of oxygen was only about 40%. The synergistic mechanism of oxygen nanobubble/photolysis process was attributed that oxygen nanobubbles provided both dissolved oxygen and ROS to the reaction system, and during the photoreaction process, oxygen consumption facilitated the nanobubble burst, thereby promoting the generation of ROS of which ·OH played a dominant role in the photodegradation of oxytetracycline (Wang et al. 2020).

### Ozone-nanobubble based advanced oxidation

Advanced oxidation technology based on ozone nanobubbles can remove pollutants either through direct reaction of the compounds with ozone molecules or oxidation by ·OH, which is generated from the decomposition of ozone and bubble collapse. Both simultaneously promote the efficiency of oxidative degradation (Khuntia et al. 2015), although the latter plays a predominant role during the process because the standard oxidation potential of ozone (2.07 V) is lower than that of ·OH (2.80 V). In ozone-nanobubble based advanced oxidation processes (AOPs), enhancing both types of reaction mechanisms is essential to increase the overall oxidation efficiency. Different studies have reported that ozonation and ozone-based AOPs are affected by water properties, such as pH, concentration and type of organic matter. Regarding pH, it is known that the decomposition of ozone is accelerated when the pH or concentration of OH^−^ increase, which facilitates the chain reaction resulting in the formation of ·OH (Tomiyasu et al. 1985). Lucas et al. (2009) studied the degradation of organic matter involved in winery wastewater and found that the reduction rate of the chemical oxygen demand at alkaline and neutral pHs was more accelerated than at acidic pHs due to the formation of radical species from the decomposition of ozone (Lucas et al. 2009). Similarly, the pH of the medium strongly influences the stability of the bulk nanobubbles as discussed in previous section. Interestingly, the reaction between ozone and water molecules (O_3_  +  H_2_O  →  O_2_  +  OH^−^  +  OH^−^) increases OH^−^ ions, which are responsible for producing more negatively charged nanobubbles, resulting in considerable delivery efficiency of ozone molecules for the oxidation process. The restricted efficiency of ozone is due to its relatively low solubility and rapid decomposition in the aqueous phase. Nanobubbles as ozone delivery carrier enhance ozone concentration and prolong the ozone half-life in solution, leading to reduction of ozone wastage and increment reaction rates with contaminants. Batagoda et al. (2019) compared the life of ozone bubbles produced by a regular diffuser and a nano-diffuser, showing that after 1 h of stabilisation, nano-ozone bubbles retained ozone in water for approximately four times longer than those produced using a regular diffuser (Batagoda et al. 2019). Hewage et al. (2020) designed an ultrasound and ozone nanobubble-coupled method to remediate the organic pollutants in sediments, p-terphenyl as simulated contaminant, of which the ultrasound was used to desorb the contaminants from sediments to suspension and then the pollutants were degraded by ozone. The results showed that ozone concentration delivered by nanobubbles was much higher than that with regular diffuser, concurrently, the reduction rate of ozone concentration of the former was far slower than the latter. The p-terphenyl treatment efficiency with ultrasound but without ozone nanobubbles was only 76.7%, while that of 91.5% was achieved when combined the two steps (Hewage et al. 2020). On the other hand, the possibility of improving the oxidative efficiency of an ozone-based water treatment unit depends largely on the ·OH exposure. Terfasa (2017) reported that a reduction in bubble size to the nanoscale resulted in an increase in the concentration of ·OH by a minimum of 3.5-fold when compared to the microbubble system, even in acidic media (Terfasa 2017), promoting the oxidation of small molecular organic matter such as olefins, having C = C bonds to CO_2_ and converting large molecular content to small sizes for subsequent combined biological processes such as MBRs, if necessary. 1,4-Dioxane, which has low microbial degradability and shows potential carcinogenicity to humans, was oxidised by the ozone-nanobubble system; the concentration decreased in the exponential decay model and dropped from 0.33 mg/L to the detection limit (0.005 mg/L) in 2–3 h, which was ascribed to the large amount of ·OH generated (Maie et al. 2020). As we can see, different types of gas source in bulk nanobubble-based system all get impressive removal efficiency of various organic pollutants by advanced oxidation which are briefly shown in Table 1.

**Table 1:** Typical researches of removal efficiency of organic pollutants by bulk nanobubble-based advanced oxidation

Organic pollutant	Technology	Gas source	Removal rate	Mechanism	Reference
Dark green Rit dye	Nanobubbles alone; ultrasonic nanobubbles; nanobubbles/H_2_O_2_	Air	90%	Surface charge attraction; ·OH, HO_2_·, O_2_·	(Bui and Han 2020)
Sodium dodecyl benzene sulfonate	Nanobubbles/vacuum ultraviolet system	Oxygen	99.8%	·OH	(Tasaki et al. 2009)
Oxytetracycline	Nanobubbles with photodegradation	Oxygen	98%	·OH	(Wang et al. 2020)
1,4-Dioxane	Nanobubble system	Ozone	98.5%	·OH	(Maie et al. 2020)

### Ozone-integrated biological technologies

Ozonation hybrids with sustainable biological methods, as discussed in the previous section, such as biological activated carbon and membrane bioreactors, are powerful for reducing recalcitrant chemicals and removing toxic pollutants. Wang et al. (2019) explored the advanced treatment of bio-treated dyeing and finishing wastewater (BDFW) in textile industries through the O_3_-BAC system. The dissolved organic carbon and chemical oxygen demand removal rates were only 6% and 16.5%, respectively by individual ozonation, and 33% and 40% by BAC alone, while 43% and 45.8% reduction were achieved by the O_3_-BAC combined system, respectively, the addition of ozone had no obvious improvement for removal efficiency (Wang et al. 2019). BAC alone preferentially degrades biodegradable fractions such as small molecular acids, ketones, alcohols, and halogen-containing compounds but rarely degrades fluorophores, whereas ozonation is capable to transfer hydrophobic or hydrophilic protein-like fluorophores to less fluorescent transformation products that are more susceptible to biodegradation via BAC treatment, which explains the superior performance of the O_3_-BAC combination system. Hu et al. (2021) integrated ozone with co-immobilised microalgae-activated sludge bacterial biodegradation to remove organic compounds in meat processing wastewater and reported that bacterial growth was encouraged in ozone-pretreated wastewater (7.1–8.1 log CFU/mL) compared with the non-pretreated control (6.0 log CFU/mL). This was found to be due to the enhanced biodegradability of contaminants in the same way as algal biomass growth; following pre-treatment with a favourable concentration of ozone, the soluble chemical oxygen demand achieved a removal rate of 60.1% by microalgal biotreatment, while it is only 34.4% in control (non-ozonated wastewater), confirming the synergistic effects of these two technologies (Hu et al. 2021). Nonetheless, the removal rate remains restricted and dissatisfactory; thus, it is of great need to investigate whether bulk nanobubbles, which are beneficial to individual ozonation or biological treatment methods, can facilitate hybrid treatment efficiency.

## Conclusions

**Fig. 1:**
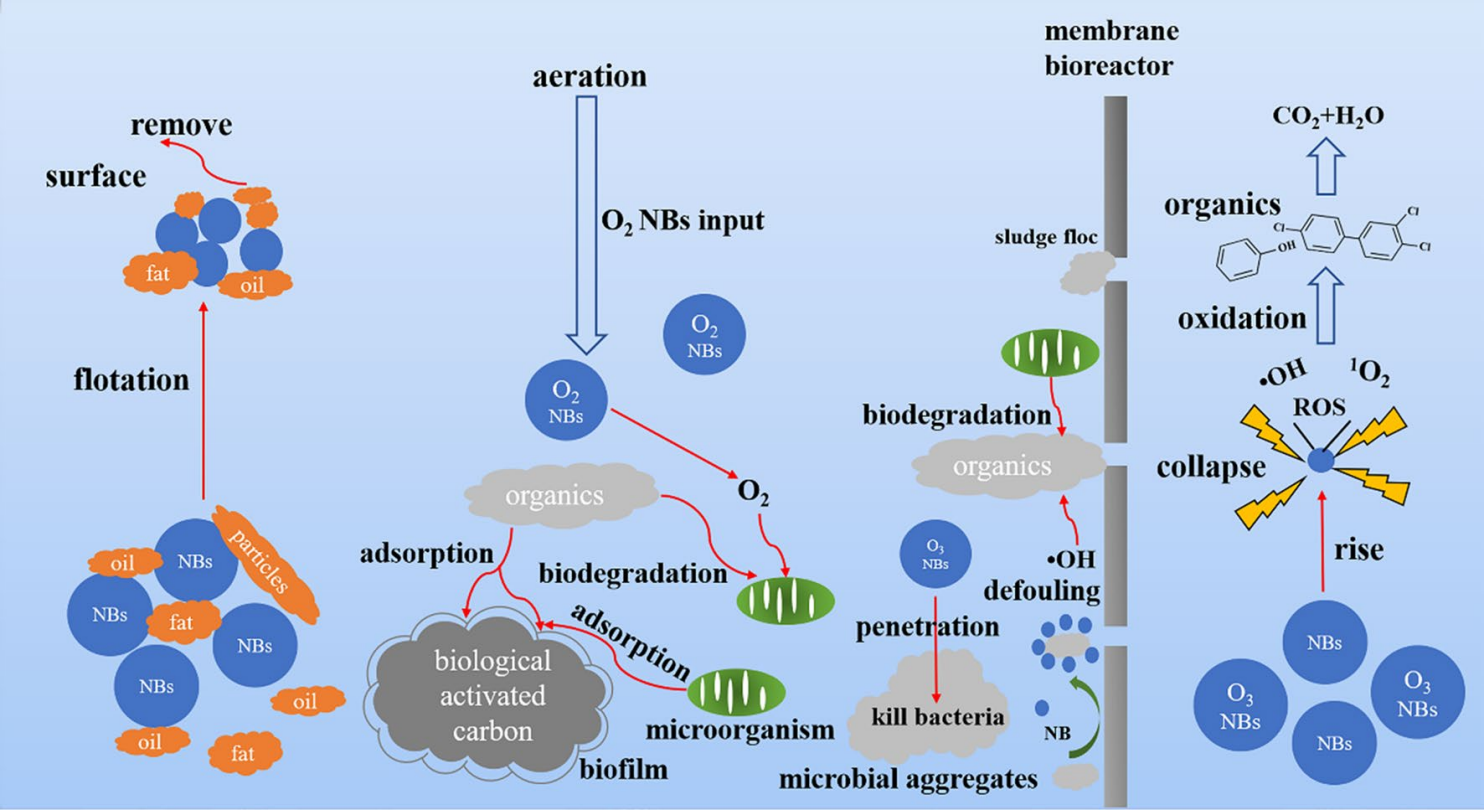
The pathways for removing organic pollutants by bulk nanobubble-integrated technologies in wastewater

Although the initial proposal of bulk nanobubbles was controversial, their existence in water has since been proven experimentally owing to the continuous exponential increase in research efforts in recent years. It is clear that the unique features of nanobubbles such as high stability, long lifetimes, large surface-volume ratio, high mass transfer efficiency, and capability to generate free radicals, can provide various means to improve conventional technologies in the water treatment field. For future perspectives, considering the wide applications of nanobubbles in order to further exploit their usage and gain a better in depth understanding of the parameters that having profound impacts are crucial. Nanobubbles have significant potential as a new environmentally friendly method to remove organic compounds as shown in Fig. 1 through their small size and existence of a surface charge, which effectively improve the air flotation process to separate suspensions; the large specific surface area and durability of nanobubbles allow the enhancement of the oxygen mass transfer efficiency to promote aeration for aerobic microorganisms to biodegrade organics and decrease the production of excess sludge in the activated sludge process, and alleviate fouling on the membrane of MBRs; moreover, the ability to generate free radicals with considerable oxidation function promotes the degradation of organic compounds. In this regard, this technology has significant value for treating wastewater containing organic pollutants.

Nevertheless, further investigation of bulk nanobubbles is required. Only by solving these challenging issues can we make a breakthrough to provide a solid theoretical basis for the industrial scale operations of bulk nanobubbles. The topics of subsequent research can be determined based on the following issues:

(1) Many researchers have proposed that a large amount of active oxygen will be produced when nanobubbles rupture, and the existence of ·OH during the application of nanobubbles has been proven. However, other ROS species such as O_2_·^−^and ^1^O_2_ have not been confirmed through mature techniques; thus, their effectiveness has yet to be evaluated, and the interpretation of ROS generation remains controversial. Additionally, various reports verify that ROS concentration has different influences including promotion or toxicity to plants, but the synergistic and antagonistic interaction of ROS with microbial activities during application of nanobubble techniques are uncovered.

(2) The advantages of bulk nanobubbles in aeration during the MBR biodegradation process have been highlighted. Nevertheless, few studies have focused on the probable mechanism of nanobubbles in membrane cleaning, which leads to reduce the requirement of chemicals such as antiscalants, prolong the membrane lifetime, and decrease operational expenses.

(3) Advanced oxidation processes which mainly depend on ·OH, such as ozonation, have been integrated with MBR systems for the treatment of various wastewaters, including oils and pharmaceuticals, dyeing or textile wastewater, municipal effluent, and domestic and industrial wastewater. Further investigations are required to elucidate the influence of bulk nanobubbles on the treatment efficiency of these hybrid systems.

(4) Organic compounds, which cause taste and odour problems, such as 2-methylisopropanol and geosmin, generated from microorganisms that persistently exist in tap water even after it has been treated by water plants, have attracted great attention from the public in recent years. Thus, new effective methods are of urgently required, and how to combine bulk nanobubbles with biological activated carbon to adsorb and oxidise these taste and odor compounds via ·OH generated by bulk nanobubbles, deserves more attention.

(5) Some investigations have indicated nanobubbles consume lower energy than other methods, but there is no specific data for reliable quantification. A comprehensive cost-benefit analysis should consist of the whole processes of the nanobubble treatment system, including the production of devices, operation, maintenance, disposal, etc. The production of devices which are low-cost competitive devices that show superior performance and can be used in practical engineering is of great importance.

(6) So far, research on the application of bulk nanobubbles to the treatment of organic pollutants is limited. The existence of various substances in wastewater and the influence of the operating conditions on the treatment need to be investigated, and the process of conversion of organic matter remains to be discussed.

## Data Availability

Not applicable.
